# Local-regional control according to surrogate markers of breast cancer subtypes and response to neoadjuvant chemotherapy in breast cancer patients undergoing breast conserving therapy

**DOI:** 10.1186/bcr3198

**Published:** 2012-05-23

**Authors:** Abigail S Caudle, Tse-Kuan Yu, Susan L Tucker, Isabelle Bedrosian, Jennifer K Litton, Ana M Gonzalez-Angulo, Karen Hoffman, Funda Meric-Bernstam, Kelly K Hunt, Thomas A Buchholz, Elizabeth A Mittendorf

**Affiliations:** 1 Department of Surgical Oncology, The University of Texas MD Anderson Cancer Center, 1400 Hermann Pressler Drive, Unit 1484, Houston, TX 77030, USA; 2 Department of Radiation Oncology, The University of Texas MD Anderson Cancer Center, 1515 Holcombe Blvd, Unit 0097, Houston, TX 77030, USA; 3 Department of Bioinformatics and Computational Biology, The University of Texas MD Anderson Cancer Center, 1515 Holcombe Blvd, Unit 1410, Houston, TX 77030, USA; 4 Department of Breast Medical Oncology, The University of Texas MD Anderson Cancer Center, 1155 Hermann Pressler Drive, Unit 1354, Houston, TX 77030, USA

## Abstract

**Introduction:**

Breast cancers of different molecular subtypes have different survival rates. The goal of this study was to identify patients at high risk for local-regional recurrence according to response to neoadjuvant chemotherapy and surrogate markers of molecular subtypes in patients undergoing breast conserving therapy (BCT).

**Methods:**

Clinicopathologic data from 595 breast cancer patients who received neoadjuvant chemotherapy and BCT from 1997 to 2005 were identified. Estrogen receptor (ER), progesterone receptor (PR) and human epidermal growth factor receptor 2 (HER2) expression determined by immunohistochemistry were used to construct the following subtypes: ER+ or PR+ and HER2- (hormone receptor (HR)+/HER2-; 52%), ER+ or PR+ and HER2+ (HR+/HER2+; 9%), ER- and PR- and HER2+ (HR-/HER2+; 7%) and ER- and PR- and HER2- (HR-/HER2-; 32%). Actuarial rates were calculated using the Kaplan-Meier method and compared using the log-rank test. Cox proportional hazards models were used for multivariate analysis (MVA).

**Results:**

After a median follow-up of 64 months, the five-year local-regional recurrence (LRR)-free survival rate for all patients was 93.8%. The five-year LRR-free survival rates varied by subtype: HR+/HER2- 97.0%, HR+/HER2+ 95.9%, HR-/HER2+ 86.5% and HR-/HER2- 89.5% (*P *= 0.001). In addition to subtype, clinical stage III disease (90% vs. 95% for I/II, *P *= 0.05), high nuclear grade (92% vs. 97% with low/intermediate grade, *P *= 0.03), presence of lymphovascular invasion (LVI) (89% vs. 95% in those without LVI, *P *= 0.02) and four or more positive lymph nodes on pathologic examination (87% vs. 95% with zero to three positive lymph nodes, *P *= 0.03) were associated with lower five-year LRR-free survival on univariate analysis. On MVA, HR-/HER2+ and HR-/HER2- subtypes and disease in four or more lymph nodes were associated with decreased LRR-free survival. A pathologic complete response (pCR) was associated with improved LRR-free survival.

**Conclusions:**

Patients with HR+/HER2- and HR+/HER2+ subtypes had excellent LRR-free survival regardless of tumor response to neoadjuvant chemotherapy. Patients with HR-/HER2+ and HR-/HER2- subtypes with poor response to neoadjuvant chemotherapy had worse LRR-free survival after BCT. Additional study is needed to determine the impact of trastuzumab on local-regional control in HER2+ tumors. Our data suggest that patients with HR-/HER2- subtype tumors not achieving pCR may benefit from novel strategies to improve local-regional control.

## Introduction

The local-regional management of breast cancer has evolved significantly over the past several decades. Based on data from prospective studies, breast conservation therapy (BCT), defined as lumpectomy with whole breast irradiation, is now accepted as oncologically equivalent to mastectomy in terms of overall survival (OS) [1-6]. The number of patients who are candidates for BCT has increased with the use of neoadjuvant chemotherapy, which has been shown to downsize tumors, facilitating BCT in patients that would otherwise require mastectomy if surgery were performed first [7-9]. Numerous clinical trials have investigated the impact of neoadjuvant versus adjuvant chemotherapy on survival and two meta-analyses have reported equivalent outcomes [10,11]. The use of neoadjuvant chemotherapy has also allowed insight into tumor biology and differential response to treatment. Studies have shown that estrogen receptor (ER)-negative and high-grade tumors are more likely to respond to neoadjuvant chemotherapy [12-14]. In addition, multiple previous reports have demonstrated that achieving a pCR with neoadjuvant chemotherapy is associated with improved overall survival (OS) [9,15-17]. The effects of response to neoadjuvant chemotherapy on local-regional control are less well studied.

The importance of the underlying biology of breast tumors in predicting outcomes has been demonstrated by microarray analyses that identified molecular subtypes. The subtypes were initially defined by gene expression profiles that broadly divided breast tumors into subgroups, including: ER positive/luminal-like, basal-like, HER2 positive and normal breast [18]. Subsequent studies demonstrated that the ER positive/luminal-like group could be further refined into two subgroups; luminal A and luminal B with luminal A having greater expression of ER-related genes and luminal B having higher expression of proliferative genes [19,20]. These classifications were determined to be clinically relevant when it was demonstrated that there were significant differences in survival based on the subtype. ER/luminal tumors had longer disease-free and OS while basal-like and HER2 subtypes had worse outcomes [19].

Although determination of molecular subtype may be the most accurate way to evaluate breast cancers, such molecular profiling is currently not feasible for routine clinical care due to the time, cost and resources required to perform the analysis. In addition, it has not yet been demonstrated that decisions regarding local-regional or systemic treatment options should be made based on molecular subtyping. Therefore, clinicians have used hormone receptor and HER2 status, which are routinely provided in a patient's pathology report, to group tumors into constructed subtypes [21,22]. These constructed subtypes, which have been approximated as luminal A (ER+ or progesterone receptor (PR)+, HER2-), luminal B (ER+ or PR+, HER2+), HER2 (ER-, PR-, HER2+) and basal (ER-, PR-, HER2-), have been shown to approximate the molecular subtype signatures [23]. In a study evaluating almost 800 patients, Nguyen *et al. *reported that local recurrence rates following BCT in patients undergoing surgery as a first intervention varied according to subtype approximated using ER, PR and HER2 status [24]. Because neoadjuvant chemotherapy is increasingly used in breast cancer patients to facilitate BCT and because response to neoadjuvant chemotherapy has been shown to provide prognostic information, we undertook the current study to determine if categorizing patients into subgroups using ER, PR and HER2 status could predict response to neoadjuvant chemotherapy and identify patients at high risk for local-regional recurrence (LRR) following neoadjuvant chemotherapy and BCT.

## Materials and methods

### Patient population

A prospectively maintained database was used to identify patients with non-metastatic breast cancer who received neoadjuvant chemotherapy followed by BCT from 1997 to 2005. The study was approved by the University of Texas MD Anderson Cancer Center Institutional Review Board. Clinicopathologic data were recorded, including: clinical T and N stage according to the sixth edition of the American Joint Committee on Cancer staging guidelines, pathologic tumor size, number of lymph nodes identified pathologically, Black's modified nuclear grade, presence of lymphovascular invasion (LVI), ER, PR and HER2 status. Clinical T and N stage were determined at presentation by physical examination, mammography and ultrasound (US) of the breast and regional nodal basins. Lymph nodes appearing abnormal on US were routinely evaluated by fine needle aspiration biopsy [25]. For hormone receptor status, > 10% staining of cells by immunohistochemistry (IHC) was considered positive. Tumors were considered HER2-positive if they were 3+ by IHC or demonstrated gene amplification by fluorescence *in situ *hybridization. A pCR was defined as no residual invasive disease in the breast or axilla.

The study population of 595 patients was made up of patients for whom ER, PR and HER2 status was known. ER and PR status were categorized as hormone receptor (HR) positive if ER or PR staining was positive and HR negative if ER and PR staining were negative. These patients were then categorized based on their constructed subtype as follows: HR+/HER2- (ER+ or PR+ and HER2-), HR+/HER2+ (ER+ or PR+ and HER2+), HR-/HER2+ (ER- and PR- and HER2+) and HR-/HER2- (ER- and PR- and HER2-).

### Treatment

All patients received neoadjuvant chemotherapy consisting of an anthracycline (98%), taxane (84%) or a combination of the two. Because this study predated the routine use of neoadjuvant trastuzumab therapy, patients receiving neoadjuvant trastuzumab were excluded. Following neoadjuvant chemotherapy, all patients underwent BCT, including lumpectomy, axillary node evaluation and whole breast irradiation. At MD Anderson we have developed a standard approach whereby all patients undergo imaging before and after chemotherapy (diagnostic mammography and ultrasound). The ultrasound evaluation includes the breast and regional nodal basins. Any suspicious appearing lymph nodes are confirmed to be positive for metastasis by fine needle aspiration biopsy. A marking clip is placed at the tumor site early in the treatment course to facilitate resection of the primary tumor bed in case of complete radiographic response. Following neoadjuvant chemotherapy, any residual radiographic abnormality and the clip are targeted for resection along with at least a 2 mm margin of normal tissue. In most cases, we do not attempt to excise the entire pre-chemotherapy tumor volume [26-28]. Patients with positive margins at initial attempt at BCT who were then converted to mastectomy were excluded from this study. For patients presenting with clinically node negative disease, axillary evaluation consisted of sentinel lymph node (SLN) dissection with completion axillary lymph node dissection (ALND) performed when the SLN showed metastatic disease. Patients presenting with clinically node positive disease underwent ALND. Radiation included external-beam therapy to the whole breast with tangential fields. Standard treatment included a 50 Gy median dose to the breast delivered over five weeks in 25 fractions followed by a boost to the tumor bed (10 Gy median dose). Regional nodal irradiation (RNI) was administered at the discretion of the radiation oncologist and was generally considered for patients with clinical stage III disease, those with residual positive lymph nodes identified pathologically and selectively for other indications to include young age, residual tumor > 2 cm, LVI and pretreatment extent of disease on US. Patients with hormone receptor positive disease were routinely offered adjuvant endocrine therapy.

### Endpoints and statistical methods

The primary endpoint was LRR defined as disease recurrence in the ipsilateral breast or the axillary, supraclavicular, infraclavicular or internal mammary lymph nodes. All LRRs were considered events regardless of whether they were the first site of failure or occurred with or after distant metastasis. Patients who did not experience a LRR were censored at last follow-up or at the time of death.

Distributions of clinical factors between groups were compared using the Kruskal-Wallis test for continuous variables and chi-squared test for categorical variables. Actuarial rates of LRR were calculated using the Kaplan-Meier method and differences between groups were compared using the log-rank test. Multivariate analyses were performed using a Cox proportional hazards model. All calculations were performed with Stata software (Stata/SE 11; Stata Corp., College Station, TX, USA). Two-tailed *P-*values ≤ 0.05 were considered statistically significant.

## Results

The study population consisted of 595 patients who received neoadjuvant chemotherapy and then underwent BCT; 309 (52%) categorized as HR+/HER2-, 51 (9%) HR+/HER2+, 42 (7%) HR-/HER2+, and 193 (32%) HR-/HER2-. Table 1 lists clinicopathologic characteristics by constructed subtype. There were significant differences in the distribution of clinical N stage (*P *= 0.04) between the subtypes, driven largely by HR-/HER2+ patients being less likely to have clinical N0 disease. There was also a significant difference (*P *< 0.001) with respect to nuclear grade with patients in the HR-/HER2+ and HR-/HER2- groups having a higher percentage of patients with grade 3 disease. When evaluating response to neoadjuvant chemotherapy, we noted a difference (*P *< 0.001) in pCR rates with a lower percentage of patients in the HR+/HER2- (9%) and HR+/HER2+ (18%) subgroups achieving a pCR compared with patients in the HR-/HER2+ (36%) and HR-/HER2- (38%) subgroups. Patients with HR-/HER2+ and HR-/HER2-tumors had smaller pathologic tumor sizes and a greater proportion had fewer than four positive lymph nodes identified at pathologic evaluation after surgery.

**Table 1:** Clinicopathologic characteristics by constructed molecular subtype

Characteristic	HR+/HER2- (*n *= 309)	HR+/HER2+ (*n *= 51)	HR-/HER2+ (*n *= 42)	HR-/HER2- (*n *= 193)	* *P-value*
Age, years					0.28
Median (range)	51 (29 to 83)	51 (27 to 72)	49 (26 to 65)	49 (27 to 76)	

Clinical T-stage					0.09
T0	1 (0.3%)	0	0	0	
T1	65 (21%)	5 (10%)	6 (14%)	19 (10%)	
T2	207 (67%)	36 (70%)	33 (79%)	140 (73%)	
T3	25 (8%)	5 (10%)	3 (7%)	22 (11%)	
T4	10 (3%)	5 (10%)	0	12 (6%)	
Tx	1 (0.3%)	0	0	0	

Clinical N-stage					0.03
N0	167 (54%)	29 (57%)	11 (26%)	101 (52%)	
N1	110 (36%)	15 (29%)	23 (55%)	62 (32%)	
N2	12 (4%)	1 (2%)	4 (9.5%)	9 (5%)	
N3	20 (6%)	6 (12%)	4 (9.5%)	21 (11%)	

Clinical stage					0.18
I	26 (8%)	4 (8%)	2 (5%)	8 (4%)	
II	234 (76%)	35 (69%)	30 (71%)	137 (71%)	
II	50 (16%)	12 (23%)	10 (24%)	48 (25%)	

Nuclear grade					< 0.001
1	15 (5%)	1 (2%)	0	0	
2	144 (47%)	14 (28%)	3 (7%)	21 (11%)	
3	149 (48%)	36 (70%)	39 (93%)	172 (89%)	
Unknown	1 (0.3%)	0	0	0	

LVI					0.47
Yes	55 (18%)	5 (10%)	7 (17%)	28 (15%)	
No	254 (82%)	46 (90%)	35 (83%)	165 (85%)	

Pathologic tumor size, cm					< 0.001
Median (range)	1.5 (0 to 9)	1.1 (0 to 4)	0.1 (0 to 8)	0.3 (0 to 5.5)	

Number positive lymph nodes					
0	152 (49%)	34 (67%)	27 (64%)	149 (77%)	< 0.001
1-3	103 (33%)	9 (18%)	11 (26%)	32 (17%)	
≥ 4	53 (17%)	6 (12%)	4 (10%)	12 (6%)	
Unknown	1 (0.3%)	2 (3%)	0	0	

Number lymph nodes sampled					0.04
Median (range)	12 (0 to 40)	5 (0 to 27)	13 (1 to 37)	9 (1 to 30)	

pCR					< 0.001
Yes	27 (9%)	9 (18%)	15 (36%)	73 (38%)	
No	282 (91%)	42 (18%)	27 (64%)	120 (62%)	

*The Kruskal-Wallis test was used for age (continuous variable). All other *P-*values were determined using the χ^2 ^test for equality of distributions. LVI, lymphovascular invasion; pCR, pathologic complete response

**Figure 1:**
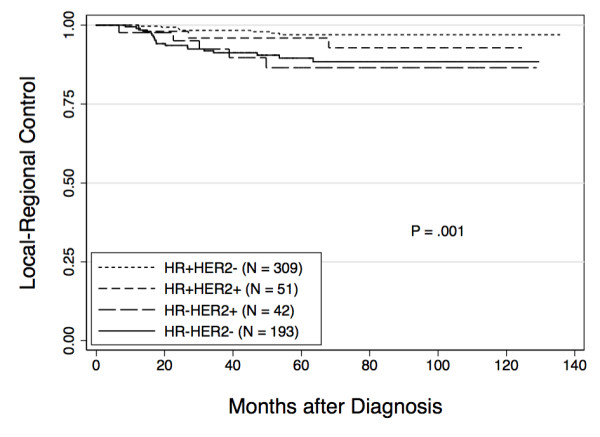
**Local-regional recurrence free survival by constructed subtype**. Actuarial rates of local-regional recurrence were calculated using the Kaplan-Meier method and differences between groups were compared using the log-rank test.

Median follow-up for the entire study population was 64 months (range 4 to 136 months). There were 24 local recurrences and 11 regional recurrences and the five-year LRR-free survival rate for the entire population was 93.8%. The five-year LRR-free survival rate was higher for HR+/HER2- and HR+/HER2+ patients when compared to HR-/HER2+ or HR-/HER2- patients (97.0%, 95.9%, 86.5%, 89.5%, respectively, *P *= 0.001) (Figure 1). The distribution of local versus regional recurrences by subtype is shown in Table 2. The five-year overall survival (OS) rate for the entire population was 88.2%. The five-year OS rates by subtype were: HR+/HER2- 92.5%, HR+/HER2+ 85.8%, HR-/HER2+ 84.4%, and HR-/HER2- 83.0% (*P *= 0.008).

**Table 2:** Distribution of local-regional recurrences by constructed molecular subtype

	HR+/HER2- *N *= 309	HR+/HER2+ *N *= 51	HR-/HER2+ *N *= 42	HR-/HER2- *N *= 193
Local	4	2	4	14

Regional	4	1	1	5

In addition to constructed subtype, we also evaluated the effect of other clinical and pathologic features on LRR-free survival rates. As shown in Table 3 clinical stage III disease, four or more positive lymph nodes, presence of LVI and high nuclear grade were all significantly associated with lower LRR-free survival rates on univariate analysis. In order to determine the impact of these factors based on subtype, analyses were repeated with patients divided by constructed subtype (Table 4). Among patients who did not achieve a pCR or who had four or more positive lymph nodes, those who were HR-/HER2+ and HR-/HER2- had decreased five-year LRR-free survival, while HR+/HER2-and HR+/HER2+ patients continued to have high rates of LRR-free survival. For example, the five-year LRR-free survival rate for patients categorized as HR-/HER2-that achieved a pCR was 99%, whereas in those who did not achieve a pCR, the five-year LRR-free survival rate was 84%. In contrast, for patients with HR+/HER2-disease, the five-year LRR-free survival rate was 96% for those who achieved a pCR and 97% for those who did not. Similarly, for patients that had four or more positive lymph nodes, the five-year LRR-free survival rate for patients with HR-/HER2- tumors was 56%, versus 91% if they had zero to three positive lymph nodes. For patients with HR+/HER2- disease, the five-year LRR-free survival rates were 93% and 98%, respectively, for those who had four or more positive lymph nodes versus those who had zero to three positive nodes.

**Table 3:** Five-year actuarial rates of local-regional control according to clinical and pathological disease status

Factor	5-year LRC rate	*P-value*
Clinical Stage		
I/II (*n *= 475)	95%	0.05
III (*n *= 120)	90%	

Number positive nodes*		
0 to 3 (*n *= 517)	95%	0.03
≥ 4 (*n *= 75)	87%	

pCR		
No (*n *= 471)	93%	0.06
Yes (*n *= 124)	97%	

LVI		
No (*n *= 500)	95%	0.02
Yes (*n *= 95)	89%	

Nuclear grade**		
1 or 2 (*n *= 198)	97%	0.03
3 (*n *= 396)	92%	

* In three patients the number of lymph nodes was unknown. ** In one patient, the nuclear grade was unknown. LRC, local-regional control; LVI, lymphovascular invasion; pCR, pathologic complete response

**Table 4:** Five-year actuarial rates of local-regional control according to clinical and pathologic disease status by molecular subtype

Factor	HR+/HER2- (*n *= 309)	HR+/HER2+ (*n *= 51)	HR-/HER2+ (*n *= 42)	HR-/HER2- (*n *= 193)	*P-value*
Clinical stage					
I/II (*n *= 473)	98%	95%	86%	91%	0.004
III (*n *= 120)	92%	100%	89%	84%	0.25

Number positive nodes*					
0 to 3 (*n *= 517)	98%	95%	89%	91%	0.007
≥ 4 (*n *= 75)	93%	100%	67%	56%	0.001

pCR					
No (*n *= 471)	97%	95%	83%	84%	< 001
Yes (*n *= 124)	96%	100%	92%	99%	0.62

LVI					
No (*n *= 500)	97%	95%	83%	93%	0.007
Yes (*n *= 95)	96%	100%	100%	70%	0.001

Nuclear grade**					
1 or 2 (*n *= 197)	97%	100%	67%	100%	0.009
3 (*n *= 395)	97%	94%	88%	88%	0.04

* In three patients the number of lymph nodes was unknown. ** In one patient, the nuclear grade was unknown. LRC, local-regional control; LVI, lymphovascular invasion; pCR, pathologic complete response

A multivariate analysis was performed that included constructed subtype, clinical stage, grade, presence or absence of LVI, pCR versus no pCR and number of positive lymph nodes dichotomized as zero to three versus four or more. Using HR+/HER2- as the referent, HR-/HER2+ (Hazard ratio 5.7, 95% CI: 2.0 to 16.3, *P *= 0.001) and HR-/HER2- (Hazard ratio 5.7, 95% CI: 2.6 to 112.3, *P *< 0.001) subtypes were associated with reduced LRR-free survival. Disease in four or more lymph nodes (Hazard ratio 2.9, 95% CI: 1.3 to 6.6, *P *= 0.01) was also independently associated with decreased LRR-free survival. Achieving a pCR (Hazard ratio 0.22 (0.07 to 0.74, *P *= 0.014) was associated with improved LRR-free survival (Table 5).

**Table 5:** Multivariate analysis of factors associated with local-regional control

Factor	Hazard Ratio (95% CI)	*P-value*
Subtype*		
HR-/HER2-	5.7 (2.6 to 12.3)	< 0.001
HR-/HER2+	5.7 (2.0 to 16.3)	0.001

≥ 4 positive lymph nodes	2.9 (1.3 to 6.6)	0.01

pCR	0.22 (0.07 to 0.74)	0.01

* HR+/HER2- used as referent. CI, confidence interval; pCR, pathologic complete

## Discussion

The recognition that breast cancer is a heterogeneous disease with tumors of different molecular subtypes being driven by different biologic pathways, having different response to therapy and different survival rates, has been an important advance [18,19,29]. These subtypes, including luminal A, luminal B, HER2 and basal, were initially identified using cDNA microarray analysis. Subsequently, investigators have demonstrated that constructed molecular subtypes determined using ER, PR and HER2 status corresponds with these molecular subtypes thus allowing clinicians to apply this concept to patient care and decision making [30]. In the current study, we have determined the constructed subtype for a large population of patients that received neoadjuvant chemotherapy and then underwent BCT. We found that the constructed subtype correlated both with response to neoadjuvant chemotherapy as well as LRR-free survival. Importantly, patients with HR+/HER2- and HR+/HER2+ subtypes had excellent rates of LRR-free survival regardless of tumor response to neoadjuvant chemotherapy. In contrast, in patients with HR-/HER2- subtypes, the response to neoadjuvant chemotherapy was more likely to predict LRR-free survival, with those who responded poorly to neoadjuvant chemotherapy, having worse local-regional control after BCT.

Other groups have evaluated the impact of constructed subtypes on local-regional control in different patient populations. An early study addressing this question by Nguyen *et al.*, evaluated 793 patients treated with BCT as a first intervention between 1998 and 2001 [24]. Ninety percent of patients in their series received systemic therapy. After a median follow-up of 70 months, the five-year incidence of LRR was 0.8% for what these investigators referred to as luminal A (HR+/HER2-), 1.5% for luminal B (HR+/HER2+), 8.4% for HER2 (HR-/HER2+) and 7.1% for basal (HR-/HER2-) tumors. Similar to the findings from the current study, on multivariate analysis using HR+/HER2- as the referent, the HR-/HER2+ and HR-/HER2- subtypes were associated with higher rates of LRR. A recent publication from Arvold *et al. *also evaluated differences in LRR based on constructed subtypes in a population of patients undergoing BCT [31]. In contrast to the study by Nguyen *et al.*, which was limited to patients undergoing initial surgery, and to our study, which was limited to patients receiving neoadjuvant chemotherapy, the Arvold study included BCT patients regardless of whether they received neoadjuvant chemotherapy, adjuvant chemotherapy or no chemotherapy (46% of patients did receive systemic treatment). These investigators classified patients based on receptor status as well as nuclear grade with subgroups defined as luminal A (ER or PR+, HER2 -, grade 1 to 2), luminal B (ER or PR+, HER2 -, grade 3), luminal HER2 (ER or PR+, HER2+), HER2 (ER/PR-, HER2+), and triple negative (ER/PR-, HER2-). The five-year LRR rates were 0.8% for luminal A, 2.3% for luminal B, 1.1% for luminal HER2, 10.8% for HER2 and 6.7% for triple negative [32]. In this case, using constructed molecular subtypes, in addition to grade, they were able to classify patients based on rates of local-regional control. Investigators from the University of North Carolina used constructed molecular subtypes to predict local-regional control in 149 patients treated between 1991 and 2005 [33]. Similar to our study, they included only patients who received neoadjuvant chemotherapy; however, patients could undergo BCT or mastectomy. After a median follow-up of 55 months, they reported a higher rate of LRR in patients with HR-/HER2- tumors (14%) versus HR+/HER2- (4%), HR+/HER2+ (4%), or HR-/HER2+ (5%) (*P *= 0.03). This study also predated the routine use of trastuzumab in patients with HER2-overexpressing disease. Evaluating just those patients who underwent BCT (*n *= 49), they noted that there were no LRR events in the HR+/HER2-, HR+/HER2+, or HR-/HER2+ groups while 8% of the HR-/HER2- group developed LRR (*P *= 0.99) [33]. These data are limited by the small number of patients but their findings are consistent with the current study showing excellent rates of local-regional control for patients with HR+ breast cancer.

The current study represents the largest series evaluating the effect of subtype on response to neoadjuvant chemotherapy and LRR-free survival in patients undergoing BCT. We found that patients with HR+/HER2- and HR+/HER2+ tumors had significantly lower rates of pCR (9% and 18%, respectively) compared with patients with HR-/HER2+ or HR-/HER2- tumors (36% and 38%, respectively). This is consistent with recently published data from the I-SPY 1 TRIAL, a multicenter study evaluating patients with tumors ≥ 3 cm using molecular signatures and early imaging with outcomes of pCR and recurrence free survival (RFS) [34]. In the I-SPY trial, the pCR rate was 9% in patients with HR positive tumors, which is identical to our rate for HR+/HER2- tumors. For patients with HR-/HER2- tumors, the pCR rate was 35%, comparable to our 38% rate. One difference between these studies was the pCR rate of 54% in HR-/HER2+ patients in the I-SPY 1 trial; higher than our rate of 36% but based on only 13 patients.

Guarneri *et al. *previously reported that patients with ER-negative tumors were more likely to achieve a pCR after neoadjuvant chemotherapy [14]. The study by Guarneri and the current study do have some overlap in the population of patients reported; however, the previous report included patients that underwent mastectomy as well as BCT. Guarneri noted that patients with ER-positive tumors, despite having lower rates of pCR, had better five-year overall and progression-free survival rates. We found similar results in patients with HR+/HER2- and HR+/HER2+ disease, regardless of whether they responded to neoadjuvant chemotherapy. For patients with HR+/HER2- disease who achieved a pCR, the five-year LRR-free survival rate was 96% versus 97% for those who did not have a pCR. Similarly, for patients with HR+/HER2+ disease, the five-year LRR-free survival rates were 100% and 95%, respectively for those who did and did not have a pCR. These data likely reflect both the overall favorable biology of the HR+ subtypes as well as the effectiveness of hormonal therapy in these patients. In contrast, for patients with HR-/HER2- tumors, we found a high rate of pCR (38%) and whether or not a patient achieved a pCR had significant implications for LRR-free survival rates. Patients who achieved a pCR had a five-year LRR-free survival rate of 99% versus 84% in those who did not achieve a pCR. Our data are again consistent with recently published data from the I-SPY 1 TRIAL, where they found that the association between RFS and pCR was greatest for patients who did not have HR+/HER2- tumors [34]. The use of neoadjuvant chemotherapy in patients with HR-/HER2- tumors could, therefore, be useful in helping to identify patients at higher risk of LRR. For patients with HR-/HER2- tumors who fail to achieve a pCR, particularly those with four or more positive lymph nodes identified at the time of surgery, strategies to improve local-regional control should be explored including mastectomy or the use of radio-sensitizers to enhance the effects of radiation in BCT patients. The identification and evaluation of radio-sensitizers is a relevant consideration as data from the Danish Breast Cancer Cooperative Group suggests that this phenotype may be less responsive to radiotherapy [35].

An important limitation of our study is the ability to draw meaningful conclusions for patients with HER2 positive tumors. This is because of the small number of patients included with HER2+ subtypes (*n *= 93, 51 HR+/HER2+ and 42 HR-/HER2+), as well as the fact that the study period predated the routine use of trastuzumab in either the adjuvant or neoadjuvant setting. The addition of trastuzumab to adjuvant chemotherapy has been shown to improve disease free and OS in HER2-positive patients [36-38]. The addition of trastuzumab to neoadjuvant chemotherapy regimens has had a significant impact as well. Studies looking at anthracycline-based neoadjuvant chemotherapy that would have included patients with HER2-positive tumors showed pCR rates of approximately 13% [39]. The addition of taxanes to anthracyclines improved pCR rates to 26 to 28% [39,40]. The addition of trastuzumab to neoadjuvant chemotherapy regimens for patients with HER2-positive disease has increased pCR rates to as high as 67% [41]. None of the patients with HER2+ tumors in the current study received trastuzumab as part of their neoadjuvant chemotherapy regimen and the pCR rate was 18% for the HR+/HER2+ subgroup and 36% in the HR-/HER2+ subgroup. We did not see significant differences in five-year LRR-free survival rates for patients with HER2+ tumors based on whether they achieved a pCR or not. This is likely attributable to the small number of patients. We did, however, note a difference in local-regional control in the HR-/HER2+ group based on the number of positive lymph nodes identified at the time of surgery with patients with zero to three positive lymph nodes having an 89% five-year LRR-free survival rate versus 67% for those with four or more positive nodes. Trastuzumab containing neoadjuvant chemotherapy regimens have been shown to eradicate clinically node positive disease in 74% of patients [42]. Taken together, this suggests that the use of trastuzumab-containing neoadjuvant chemotherapy regimens for patients presenting with clinically node positive, HER2-overexpressing breast cancer could provide important information regarding prognosis based on the extent of residual disease. In addition, similar to patients with HR-/HER2- tumors, failing to eradicate nodal disease with the use of trastuzumab-based neoadjuvant chemotherapy could identify a population who may benefit from radio-sensitizers to enhance the response to therapy. Future studies with a larger cohort of patients treated with more contemporary chemotherapy regimens incorporating trastuzumab will be required to better determine the effects of pCR on LRC in patients with HER2-positive disease.

## Conclusions

In summary, we have shown that constructed subtypes can predict response to neoadjuvant chemotherapy as well as LRR-free survival rates. Patients with HR positive tumors have a low risk of local-regional failure regardless of tumor response to neoadjuvant chemotherapy. In contrast, in patients with HR-/HER2- tumors, the response to therapy has important implications for the risk of LRR and may help to identify patients who may benefit from novel strategies to improve local-regional control.

## Abbreviations

ALND: axillary lymph node dissection; BCT: breast conserving therapy; ER: estrogen therapy; HER2: human epidermal growth factor receptor 2; HR: hormone receptor; IHC: immunohistochemistry; LRR: local-regional recurrence; LVI: lymphovascular invasion; MVA: multivariate analysis; OS: overall survival; pCR: pathologic complete response; PR: progesterone receptor; RFS: recurrence free survival; RNI: regional nodal irradiation; SLN: sentinel lymph node; US: ultrasound.

## Competing interests

The authors declare that they have no competing interests.

## Authors' contributions

ASC participated in the drafting of the manuscript. TKY conceived of the study, and participated in data collection and analysis. SLT participated in data analysis and drafting of the manuscript. IB, FMB, KH, KKH and TAB participated in data interpretation and critical revision of the manuscript. JKL and AMG participated in data collection and critical revision of the manuscript. EAM conceived of the study, participated in data collection and analysis, and drafting of the manuscript. All authors read and approved the final manuscript.
